# Exploring Reasons for Low Attendance of Mothers to Growth Monitoring and Promotion Program at Loka Abaya District, Southern Ethiopia: Exploratory Qualitative Study

**DOI:** 10.1155/2019/3510649

**Published:** 2019-02-24

**Authors:** Mesfin Tekle, Befikadu Tariku, Amsalu Alagaw, Eshetu Zerihun, Habtamu Wondiye Bekele

**Affiliations:** ^1^Save the Children, Addis Ababa, Ethiopia; ^2^Department of Public Health, Arba Minch University, Arba Minch, Ethiopia; ^3^School of Public Health, College of Medicine and Health Sciences, Bahir Dar University, Bahir Dar, Ethiopia

## Abstract

**Background:**

Different organizations in Ethiopia are currently working on prevention and promotion activities to fight malnutrition among children through a community-based nutrition program. One of these activities with little success is growth monitoring and promotion (GMP). Exploring the reason and better understanding of low attendance of mothers to the GMP program can help to improve the program by incorporating finding. The aim of the study was to explore reasons for low attendance to the GMP program among mothers of under-two children.

**Method:**

An exploratory qualitative study design was used to unearth reasons for low attendance of mothers with under-2-year-old child to the GMP program. In-depth interviews were carried out with 13 mothers. The data were analyzed using qualitative content analysis approach.

**Result:**

Mothers perceived that GMP is (meant) for unhealthy children (only). Again mothers mentioned community dishonor of mothers with wasted children and method of weighing a child as a community-related reason for low attendance to the GMP program. They also indicated that there was no community conversation and weak counseling of the mothers about child feeding and growth. Perception about “evil eye” was also indicated as a reason for lower attendance of the program.

**Conclusion:**

Mothers showed that there was lack of knowledge about GMP. Limited community conversation and weak counseling about child nutrition as a GMP program were explored reasons for low attendance. Other reasons mentioned by the mothers were consideration of “evil eye” and method of weighing a child. Further research is needed to explore the implementation of GMP by health workers and to evaluate the extent of the identified reasons for low attendance to the GMP program by the mother.

## 1. Introduction

Child growth and development is one of the major problems in low- and middle-income countries [1, 2]. Poor growth of children is associated with increased child mortality due to severe infections and more vulnerability to common childhood illnesses, which contributes to most of under-five child deaths [3]. Promotion of child growth is one of the health priorities in relation to control child mortality and poverty reduction [4]. The concept of growth monitoring and promotion (GMP) was introduced in the mid 1980s. It emphasized linking the results of monitoring with follow-up promotional actions in order to improve nutritional and health outcomes and subsequently reduce child deaths. The follow-up promotion of GMP includes nutrition counseling and provision of supplements, and early disease detection and treatment of child illness [5]. Improved nutritional status, increased utilization of the health services, and ultimately reduction in mortality are the main expected benefits of GMP [6]. GMP is considered as a useful intervention for better health and nutritional status of children.

Several studies have shown that there is a gap between the purpose and practice of GMP [6–8]. This can also be ascertained by the existing high prevalence of malnutrition in many developing countries [1, 9]. An exploratory study on the international panel of district medical officers regarding GMP indicated that the suboptimal function of GMP was mainly due to the lack of attendance of caregivers and their poor understanding of the concept of growth monitoring [7]. The component of GMP implementation includes the process of assessment, analysis, and counseling [6, 10]. Then, it should have to organize in a functioning health system and integrate with other health services. It has been performed by trained health workers with time for counseling and also caregivers having good access to attend or being visited at home [6].

In Ethiopia, the GMP program is one of the health extension packages. Health extension workers (HEWs) provide it with the support of the health development army (HDA). In each kebele (the lowest administrative units in Ethiopia), there is one health post and two HEWs. Health posts are designed as a center to provide preventive and basic curative service by HEWs. HEWs, in addition to perform in the health post, spend half of their time in the community for implementing health promotion works with community members [11, 12]. HDAs are volunteers who are trained by HEWs to promote healthy behaviors and strengthen and sustain community engagement and ownership [12]. The basic running cost of the health extension program is financed mainly by subnational health authorities, which makes GMP attendance free of charge for the participants [11].

Undernutrition is one of the main culprits causing high child mortality, accounting for 51% of all childhood deaths in Ethiopia [13]. Currently, different organizations in Ethiopia are working on prevention and promotion activities to fight malnutrition among children; one of the activities is GMP [8]. According to UNICEF, if the attendance of children in a community for the GMP program is below 80%, it is considered as low [14]. From 2015, Loka Abaya district's health office report, the average GMP attendance rate was 39.0% and 43.9% for the years 2013 and 2014, respectively. With the consideration of the positive effect of the GMP program on child nutrition, it is necessary to explore reasons for low attendance of mothers at the grassroots level in Ethiopia. It is also important that the problems of GMP need to be investigated in different contexts since its practice and underlying causes of the problems can differ hugely between different settings [8]. Therefore, this study was designed to explore reasons for low attendance to the GMP program among mothers having under-two children in Loka Abaya district, Southern Ethiopia.

## 2. Methods and Materials

### 2.1. Study Setting, Design, and Period

The study was conducted in the Loka Abaya District of the Sidama zone, which is located 330 kilometers south of Addis Ababa. The most common livelihood approach of the kebeles is agrarian, and only six kebeles in the district lead semipastoralist way of life. The district is divided into 26 rural and 1 urban kebeles. In the district, there are 26 health posts. Based on the district health office report, the GMP practice was low with the consideration of the UNICEF's standard. We implement a qualitative study design to explore reasons for low attendance of mothers to the GMP program in the study area. The study was carried out from March to April, 2015.

### 2.2. Recruitment of Participants

Purposive sampling method was employed to select the study participants, and we included 13 mothers. Based on the district report, kebeles were categorized as good performing and lower performing regarding the GMP program. From a kebele, one mother with under-two child was included in the study. HEWs were approached to identify mothers based on attendance to GMP sessions. The HEWs at the kebele with lower performance identified one mother who did not bring their children to the GMP site for more than eight months. In addition, HEWs from good performing kebele identified one mother who attended GMP consistently. Based on this method, eleven mothers from low performing and two mothers from better performing kebeles were included in the study. After identification of the mothers, the interviewer approached the mother for the interview.

### 2.3. Data Collection Tools and Procedures

In-depth interview guide to explore reasons of mothers for low attendance to the GMP program was used. A comfortable, private room, free from interruptions at their kebele, was used for the in-depth interview, and it takes between 45 and 60 minutes. One of the study authors (H.W.), who have a Master of Public Health specialty in Health Education and Promotion and have experience for more than 4 years, carried out the interviews. During the interview, the interviewer takes note and sets the tap record. The questions were categorized into three broad categories: experience of GMP, GMP attendance, and overall implementation of GMP by the provider.

### 2.4. Data Quality Assurance

A qualified interviewer (Master in Public Health) used to collect the data. He had training on qualitative data and previous experience of qualitative research [15]. Only one interview was done in a day with a view to attain quality in data collection. The interview was carried at their respective kebeles. Another investigator (M.T.) separately reviews the transcription. Attempt was also made to ensure data quality by purposefully sampling (criterion based and heterogeneity purposive sampling) to have representative samples. In addition to include both frequent and less-frequent attending mothers, both from agrarian and semipastoralist kebeles were included. This helped to involve both hard and easy to access study participants in the district.

### 2.5. Data Analysis

Audio recorded was transcribed verbatim. ATLAS.ti7 software was used to structure and manage the data. The data analysis was undertaken using qualitative content analysis approach in three stages: open coding, creating categories and subcategories, and abstraction/theoretical coding. The transcripts were inductively analyzed line by line in the open coding, and numerous codes were developed from the data. Subsequently, the open codes were clustered into categories and subcategories. The relationship between the core category and its subcategories was determined to formulate the general description of the research topic. The abstraction process was continued as far reasonable as possible. Analysis was also done on each interview until the core category emerged as a main concern. Once the core category generated, the sampling became selective in line with this category. Subcategories and their properties were developed using a constant comparison of the data as a whole.

### 2.6. Ethical Considerations

Ethical approval was secured from the Ethical Review Board of Addis Continental Institute of Public Health and the Institutional Research Ethics Review Committee of Arba Minch University. Written consent was obtained from each study participant after description of the objective and method of data collection. To maintain the confidentiality of the participants, transcription was done by anonymizing the identification of the individuals by removing the name and kebele (place).

## 3. Results

Thirteen in-depth interviews with mothers of under-two children and with an average of 17 pages transcription were undertaken. The characteristics of the mothers are presented in Table 1.

With the consideration of reasons for lower attendance of mothers to the GMP program, the study resulted in identifying the reasons related with five areas: individual and family; community; institution; livelihood; and culture related. Under each generic category, some subcategories, which were reported from mothers, are presented in detail.

### 3.1. Individuals' Understanding and Influence by the Family

Mothers mentioned that they understood GMP as being used only for unhealthy (especially wasted) children. If their children are healthy and well-fed, they did not want to attend the GMP program. In addition, if the mother perceived that she knew appropriate feeding practice, there was no need to attend the program. A 22-year-old mother said, “My child is healthy; I think it is not appropriate to take healthy (not wasted) child to health facility for GMP session.” Similarly, a 35-year-old mother also said, “The reason behind not to bring my child to GMP session is, first of all, he is not wasted as others; I'm trying to do my best for him. So, what is the importance of hanging up my children in front of people? I'm giving good care, so there is no need to attend GMP session.” A 20-year-old mother also said, “Even if I have no knowledge about child weighting measurement, I have the knowledge about child feeding practices. I did not add any value to a child by taking the child to GMP session.”

There was an expectation of direct benefit by the mothers during the visit of GMP sessions. When they realized that there was no direct benefit like “food containing vitamin in it,” they stopped attending the GMP session. For instance, a 20-year-old mother said, “I stopped attending GMP because HEWs stopped giving the foods that contain vitamin in it.” In the same manner, another 20-year-old mother also reported that “There was nothing given for us at the health post. So, taking my child to the GMP session does not add any value.”

According to the mothers, there were different types of influences by their husbands regarding the attendance of the GMP program. Generally, mothers indicated that their husbands were against, neutral, or supportive for the attendance of GMP sessions. A 35-year-old mother described that her husband does not allow her to attend GMP sessions. He claims that his child should not be weighed with poor family's children as a poor. Similarly, a 20-year-old mother also mentioned about discouraging attitude of her husband. She said, “My husband doesn't encourage me to attend GMP sessions. Because he heard from neighbors about the disadvantages of weighting the baby by a bag.” In another way, a 25-year-old mother mentioned about the neutrality of her husband in this regard. She said, “My husband neither supports nor opposes attending GMP sessions. Giving care for my child is my own deal.” In opposite to the aforementioned, a 20-year-old mother mentioned that her husband is an educated man and give support to her to take and check the growth of their child. She said, “My husband is happy and supports me in order to take the child to the GMP sessions….”

### 3.2. Influence by the Community

Mothers reported that others in the village influenced them regarding bringing their children to the GMP session. Perceived dishonor based on the finding of the measurement, weighing method used which is similar with weighing of goods, and exposure to ‘bad air' when cloth was removed were the reasons that used to influence. A mother of 25 years old also mentioned a fear of dishonor from community members. She said, “Weighing my child is shame for me; because people in my community dishonor me if my child is ill due to shortage of food intake (wasted).” In addition, she mentioned the risk of illness due to the measurement method. She said, “Mothers told that, because they (HEWs) weigh the child undressed; this might expose for “*bad air*” and child become ill.” Likewise, a mother of 35 years old also indicated that the pressure of some village women who advocate against weighing method as something that is used for goods not for children. She mentioned, “…they said that why we take our children to the HEWs to be hanged-up undressed, our children are not maize to be weighed….”

Mothers mentioned weak support and follow-up of HDAs both in mobilizing mothers to bring their children to GMP sessions and in strengthening the program as a whole. For instance, a mother of 20 years old said, “I do not appreciate HDA involvement in GMP because usually I see small number of mothers measuring their children at the health post. If HDAs helped HEWs, the utilization of the community would increase.” In the same way, another mother of 20 years old told the lack of due attention to the GMP program by HDAs. She said, “I didn't see anything done by HDAs in my village. I heard that HDA was organized; but in my village, they did not give any attention to the GMP program. Likewise, a mother of 28 years old described the lack of knowledge about GMP by HDAs. She said, “One to five network leaders do not have knowledge regarding GMP. They themselves need to have training about GMP.” However, one mother appreciated the advice given by HDA. She said, “…HDA also discusses the issue of our children and they give advice to attend the GMP program.”

Mothers indicated that they did not participate in community conversation regarding the nutrition status of their children and even they did not know there was a community conversation. A 20-year-old mother mentioned, “There is no community conversation in my kebele. Even I did not hear what community conversation means.” She angrily said, “Already they neglected me!” Similarly, another 20-year-old mother said “…no community conversation in my kebele (laughing). …I was not informed whether there was a community conversation in my kebele or village… That is why I was left out for any information in my kebele.” Likewise, a mother of 31 years old also reported, “I have not participated in community conversation in relation to child growth. Because I did not saw community conversation while conducted in my village.”

### 3.3. Institutional Reasons

There was a gap in communicating the GMP service appropriately for mothers. Mothers were not aware of the existence of the service, or they knew it lately. One mother, aged 28, for example, underscored information gap about GMP service by saying “…no other pivotal problems restricted my child to miss the GMP services; rather I did not aware of the service. I didn't know why HEWs hide the information regarding GMP, though; the health post was very close to my home.” Likewise, a mother of 20 years old also indicated similar information gap about GMP service for her absence from the GMP session in the earlier ages of her child. She said, “I started attending GMP session after two months of my child's age. Before that, I had no information about GMP. One day at the immunization program, the HEW told me to follow my child's growth …then onwards his weight is measured every month.”

In the previous GMP modality, HEWs with HDAs were going to nearby place in the villages and mothers will bring their child. The service was given closer to their home. Now, mothers bring their children to health post for each GMP session. Change in this modality of conducting GMP and not communicating it with the users were indicated as the reason for low attendance. A mother of 25 years old mentioned that the change of site from village to health post level was not communicated to her. She disclosed that “the reason I could not brought my child to the GMP sessions was that the HEWs stopped coming to our village to weigh our children as before. No one informed me to take my children to another place (Health Post), even the community health promoters…. No one informed me the site change.” The change in the modality of GMP service delivery was also mentioned by mothers as a contributing factor to the distance of the GMP service site. A mother of 27 years old, for example, appreciated the previous modality of the GMP service, which somehow addressed the issue of distance and encouraged regular attendance than the current setting and modality. She said, “…as before, it is better if HEW and Community Health promoters weigh near to the village we live.”

One of the components of GMP, which makes it as an interventional program, is to counsel the mothers on child's growth and feeding practice. This component was missing during sessions of GMP. A 20-year-old mother mentioned the absence of counseling by HEW after her child was weighted. She disclosed, “After measuring the weight of my child, the HEW did not give me advice about child feeding. Simply they sent me back to home without giving me any advice about child feeding.” Another mother with similar age also indicated the inconsistency in counseling. She mentioned the existence of counseling previously, which however was discontinued. She said, “We used to discuss about child feeding practices with the HEWs after my child was weighed. However, now a day we stopped the discussion and there is no counseling by the HEWs.”

### 3.4. Influence of Livelihood

In this study, mothers who practice semipastoralist mode of life move to neighboring districts in search of grass and water for their livestock in some seasons. This situation hindered mothers from regularly bringing their children to the GMP sessions. For example, a mother aged 20 said, “I took my child to GMP session only six times in a year. Because I used to travel to the place known as “*Sachchee*” (another district) for searching grass and water for the cows. So, it was difficult for me to come here to take my child to the GMP session every month.”

### 3.5. Cultural Context

“Evil eye” was one of the issues mentioned by the mothers. Exposure to others, method used for weighing the baby, and characteristics of the child were related to it. A mother, aged 20, reported that taking her child to the GMP session will expose him to “evil eye.” She said, “Taking the child to GMP session does not add any value to the child rather it exposes her to the other people's eyes.” In the same manner, another mother with the same age also indicated that removing the clothes of children in front of other people would expose the child to evil eye. She said, “I fear my child will be ill if exposed for “evil eye.” Removing the clothes of children in front of other people will expose them to other people's eyes.” Mothers also mentioned that skin color, walking status, and size (big) of the child expose the child for “evil eye.” For instance, the same mother mentioned above said, “There are culture related practices that influenced the community including me not to take the children to GMP sessions; like those mothers that have big children refused to weigh their children saying that their size will expose them to the other people's attention and which predispose them for “evil eye.” She further said, “Those children that have “red color” are highly exposed to the people's eyes… Mothers that have children with “red color” refused to measure their children in front of people.” Another mother with an age of 35 also reported not to expose children in front of people until they started walking. She said, “…it is not good to expose the child in front of people until they start walking.”

## 4. Discussion

Lack of appropriate knowledge about GMP and their indigenous understanding of child health and growth contributed to low GMP program attendance among mothers. As expressed by mothers in the study, their indigenous knowledge of child growth and feeding practices considered as adequate and used this as a ground for not regularly attending GMP sessions. As indicated in the result, mothers perceived that GMP was considered for wasted children only, which showed that preventive interventions such as GMP are not well understood by the target population as described by the study participants. A study conducted among district medical officers during international panel on GMP reported that the most common reason for irregular attendance was lack of interest, which resulted from their own criteria to evaluate the growth and health status of their children [7]. Similarly, other studies also showed a positive relationship between knowledge about GMP and increased attendance to the GMP program, which implies low GMP program attendance with lack of knowledge about it. These studies revealed that when caregivers view GMP activities as important and comprehend what takes place, they are likely to attend more often [16, 17]. Consistent with the result of this study, the study among district medical officers panel about perception of GMP indicated that mothers' indigenous knowledge about growth and health of their children contributed to low attendance in GMP and other health services. It showed that when mothers think that their children are in good health, which commonly defined by their ability to run, gambol, etc., mothers did not consider bringing for GMP [7].

This study showed that husbands' encouragement to GMP attendance was uncommon; it was discouraging, neutral, or supportive. A qualitative study in the Northern Ethiopia indicated that without the support of the husbands, GMP is unlikely to succeed [8]. It is indicated that husbands are very influential in regard to decisions for health service utilization in developing countries [18]. Other studies, though not directly related to GMP, also reported husbands as the primary spokespersons for their family, and their say and advice are also highly valued by mothers and children in utilizing health-care services [19, 20].

Expectation of direct benefit by mothers who were required to attend the GMP program was one of the reasons, which mentioned by the mothers as a relation to GMP attendance. In the absence of “vitamin containing food” assistance, mothers decline to regularly attend GMP sessions as it was considered one of the major benefits of attending GMP. A study in Northern Ethiopia showed that the absence of direct benefits hindered mothers from regularly attending GMP sessions [8].

The community perception of GMP influences attendance of the mothers. Peers influence was indicated as a reason for low attendance of the GMP program. The common method of influence was primarily by undermining the value of the service and propagating community beliefs by linking with illness like weighing children in front of many people as causing illness to the child. These findings illuminate the extent of negative influence that put on the mothers to not to utilize GMP services. Similarly, a brief by Berkman et al. indicated that social influence (both positive and negative) is one of the proximate pathways to promote health behavior such as appropriate health service utilization [21]. For maximizing community acceptance and uptake of intervention, programs should match the community needs and value. Adaptation of the program should be considered to integrate components in a way that are acceptable and meaningful to the targets population and community [22].

The result of this study indicated the absence of well-organized and sustainable social mobilization for beneficiary mothers. Study participants did not even acknowledge the community mobilization and health promotion role of HDAs. Community conversations enable the community members to have an overall nutrition picture of their community, analyze causes of nutritional problems, and take appropriate actions [14]. The finding of this study showed that the mothers were not exposed for community conversations. The absence of this forum created the lack of potent opportunities, which could have contributed to raising the awareness of mothers and community members on the importance of GMP and motivating them to attend the program. In addition, it might solve the problem related with communication gap regarding the program. According to the report based on the district medical officers' panel, weak awareness campaigns to motivate mothers attributed to low attendance to the GMP program [7]. Similarly, a review on the role of GMP indicated that the lack of community participation was mentioned as one of the reasons for the lack of impact [10]. A study in Nigeria showed that there were improvements in the utilization of primary health services during community mobilization and advocacy in primary health-care settings [23]. These indicated that community mobilization and/or involvement have an impact for the utilization of available health services by the community [24].

Even if the health post is within the kebele, distance of the health post from the village was happened due to the change of modality in conducting GMP sessions at Health Posts (by HEWs) rather than village level (HEWs and HDAs). Similar to the report of this study, studies showed that many mothers fail to attend regularly if weighing necessitates a lengthy visit to a clinic [6, 24].

One of the main activities linked to GMP is discussing options with the caregiver and agreeing on future action, which includes among other things that tailored counseling to caregivers based on the GM result [14]. In this report, mothers mentioned that poor counseling practices by the HEWs as reflected by absence of counseling altogether and lack of tailored counseling which indicated by the mothers as a reason for irregular attendance of GMP sessions. Similarly, a study at the Lusaka district of Zambia indicated that suboptimal functioning of the facilities regarding to the GMP. It pointed that health works did not provide counseling and advice for the caregivers [16]. In addition, reviews by Ashworth et al. and Roberfroid et al. indicated that counseling is not available in many GMP programs [6, 24].

The livelihood pattern of a certain community (settled agrarian/mobile pastoralist) determines the access and utilization of health and nutrition services. In this study, the semipastoralist way of life indicated as a reason for irregular attendance of the GMP session due to the seasonal movement of families to other areas. Studies showed that mobile lifestyle of the pastoralist community was one of the major challenges for the utilization of health services [25, 26].

Cultural beliefs such as “evil eye” for well-nourished children and children with “red color” skin and feeling of shame by mothers if the child is wasted were indicated for lower attendance by the mothers. A report with one of the objectives to describe the local health beliefs and practices concerning infant and child growth indicated that part of India and Bangladesh mentioned the concept of “evil eye” with the weighing practice of the child. This concept causes some mothers to be reluctant to attend the GMP session, especially the weighing. In addition, weighing the child was considered as degrading “as if the child were a piece of meat” [27]. Interventions should be designed in ways that are acceptable and meaningful to the community [22]. It is recommended that health promotion programs should be culturally sensitive. Culturally sensitive interventions increase the receptivity and acceptance of the intervention by the community [28]. Generally, in this study, mothers mentioned some cultural beliefs such as “evil eye,” exposing to air and weighing techniques as a reason for low attendance of the GMP sessions.

### 4.1. Strength and Limitations of the Study

The findings of this study were based on in-depth interviews with 13 mothers of under-two-year children. The findings are limited to one geographical area. However, it offers an understanding of the complexity and dynamics of different reasons that contributed and interplayed for the low attendance of the GMP program by the mothers.

## 5. Conclusion

The study revealed that GMP is not well understood by the mothers, indicating that GMP is for unhealthy children as a reason for low attendance. Some of the mothers expected direct benefit from the GMP program. It was indicated that husbands' engagement for the utilization of the GMP session was pivotal. Other discouragements for the use of GMP, expectation of dishonor if the child was wasted (undernourished), and weighing method like hanging of the child were indicated as a reason for reluctant to attend GMP sessions. These facts pointed that if the program was not supported by the community and by husbands, its uptake might be affected. It was reported that there was no community conversation regarding GMP. Lack of appropriate information about GMP service and distance of the health post were institution-related reasons. Even if child feeding counseling is one of the importances of GMP, there was inconsistent regarding its practice while they were in the GMP session. Other reasons mentioned for low attendance of the GMP program were pastoral lifestyle, weighing method, and cultural issues like the consideration of “evil eye.” Beliefs and stigma are related reasons for mothers not attending the GMP program. Even if the GMP program is preventive and promotional, it is better to keep the privacy of attendants, especially while weighing the child and disclosing the nutritional status of the children. The article shows that an intervention like this one (GMP) should go hand in hand with a program empowering the community and engaging men and husbands. Further research is needed to evaluate the extent of the identified reasons for low attendance of GMP by the mother and exploration of the implementation of the GMP by the HEW at the health post.

## Figures and Tables

**Table 1:** Characteristics of mothers of under-two children involved in in-depth interview from 13 kebeles of Loka Abaya district, Southern Ethiopia, 2015 (*N* = 13).

Characteristics of participants		Frequency	Percent
Education	Illiterate	4	31
	1–4	5	38
	5–8	4	31
Age of the mothers	20–24	6	46
	25–29	4	31
	>30	3	23
Duration of GMP participation (April 2014 to March 2015)	≥10 months	1	8
	6–9 months	3	23
	<6 months	9	69
Livelihood	Agrarian	8	62
	Semipastoralist	5	38

## Data Availability

The data used to support the findings of this study are available from the corresponding author upon request.

## Abbreviations

GMP: Growth monitoring and promotion
HDA: Health development army
HEW: Health extension workers
UNICEF: The United Nations International Children's Emergency Fund.

## References

[1] Lu C., Black M. M., Richter L. M. (2016). Risk of poor development in young children in low-income and middle-income countries: an estimation and analysis at the global, regional, and country level. *Lancet Global Health*.

[2] McCoy D. C., Peet E. D., Ezzati M. (2016). Early childhood developmental status in low- and middle-income countries: national, regional, and global prevalence estimates using predictive modeling. *PLoS Medicine*.

[3] UNICEF (2009). *Tracking Progress on Child and Maternal Nutrition–A Survival and Development Priority*.

[4] Liu Q., Long Q., Garner P. (2012). *Growth Monitoring and Promotion (GMP) for Children in Low and Middle Income Countries*.

[5] Pearson R. (1995). *Thematic Evaluation of UNICEF Support to Growth Monitoring*.

[6] Ashworth A., Shrimpton R., Jamil K. (2008). Growth monitoring and promotion: review of evidence of impact. *Maternal & Child Nutrition*.

[7] Roberfroid D., Lefèvre P., Hoerée T., Kolsteren P. (2005). Perceptions of growth monitoring and promotion among an international panel of district medical officers. *Journal of Health, Population and Nutrition*.

[8] Bilal S. M., Moser A., Blanco R., Spigt M., Dinant G. J. (2014). Practices and challenges of growth monitoring and promotion in Ethiopia: a qualitative study. *Journal of Health, Population and Nutrition*.

[9] CSA (2016). *Ethiopia Demographic and Health Survey*.

[10] Mangasaryan N., Arabi M., Schultink W. (2011). Revisiting the concept of growth monitoring and its possible role in community-based nutrition programs. *Food and Nutrition Bulletin*.

[11] Bilal N. K., Herbst C. H., Zhao F., Soucat A., Lemiere C. (2011). Health extension workers in Ethiopia: improved access and coverage for the rural poor. *Yes Africa Can: Success Stories from a Dynamic Continent*.

[12] Admasu K.-B. (2016). Designing a resilient national health system in Ethiopia: the role of leadership. *Health Systems & Reform*.

[13] Government of the Federal Democratic Republic of Ethiopia (2013). *National Nutrition Programme, June 2013–June 2015*.

[14] Griffiths M., Del Rosso J. (2007). *Growth Monitoring and the Promotion of Healthy Young Child Growth: Evidence of Effectiveness and Potential to Prevent Malnutrition*.

[15] Wondiye H., Fentahun N., Limaye R. J., Kote M., Girma E. (2016). Barriers and facilitators of ART adherence in Hawassa town, Southern Ethiopia: a grounded theory approach. *Ethiopian Journal of Health Development*.

[16] Charlton K. E., Kawana B. M., Hendricks M. K. (2009). An assessment of the effectiveness of growth monitoring and promotion practices in the Lusaka district of Zambia. *Nutrition*.

[17] Gyampoh S. (2012). *Assessment of Clinic-Based Growth Monitoring and Promotion in the Accra Metropolitan Area of Ghana*.

[18] Nyandieka L. N., Njeru M. K., Ng’ang’a Z., Echoka E., Kombe Y. (2016). Male involvement in maternal health planning key to utilization of skilled birth services in Malindi Subcounty, Kenya. *Advances in Public Health*.

[19] Bredesen J. A. (2013). Women’s use of healthcare services and their perspective on healthcare utilization during pregnancy and childbirth in a small village in northern India. *American International Journal of Contemporary Research*.

[20] Fotso J. C., Fogarty L., Mohanty S. (2015). Progress towards millennium development goals 4 & 5: strengthening human resources for maternal, newborn and child health. *BMC Health Services Research*.

[21] Berkman L. F., Glass T., Brissette I., Seeman T. E., Brisette L., Seeman T. E. (2000). From social integration to health: durkheim in the new millennium. *Social Science & Medicine*.

[22] O’Connell, Boat T., Warner E. (2009). Preventing Mental, Emotional, and Behavioral Disorders among Young People: Progress and Possibilities.

[23] Adah S. O., Ogbonna C., Anga P. (2009). The impact of advocacy and community mobilization on the utilization of health services at the Comprehensive Health Centre, Gindiri. *Jos Journal of Medicine*.

[24] Roberfroid D., Kolsteren P., Hoeree T., Maire B. (2005). Do growth monitoring and promotion programs answer the performance criteria of a screening program? A critical analysis based on a systematic review. *Tropical Medicine and International Health*.

[25] Dubale T., Mariam D. H. (2007). Determinants of conventional health service utilization among pastoralists in northeast Ethiopia. *Ethiopian Journal of Health Development*.

[26] El Shiekh B., van der Kwaak A. (2015). Factors influencing the utilization of maternal health care services by nomads in Sudan. *Pastoralism*.

[27] Brownlee A. (1990). *Growth Monitoring and Promotion: the Behavioral Issues*.

[28] Resnicow K., Baranowski T., Ahluwalia J. S., Braithwaite R. L. (1999). Cultural sensitivity in public health: defined and demystified. *Ethnicity & Disease*.

